# Exercise capacity in diabetes mellitus is predicted by activity status and cardiac size rather than cardiac function: a case control study

**DOI:** 10.1186/s12933-018-0688-x

**Published:** 2018-03-23

**Authors:** Timothy J. Roberts, Andrew T. Burns, Richard J. MacIsaac, Andrew I. MacIsaac, David L. Prior, André La Gerche

**Affiliations:** 1 Department of Cardiology, St Vincent’s Hospital Melbourne, Fitzroy, Australia; 2 St Vincent’s Department of Medicine, University of Melbourne, Fitzroy, Australia; 3 Department of Endocrinology & Diabetes, St Vincent’s Hospital Melbourne, Fitzroy, Australia; 4 Department of Cardiovascular Medicine, University Hospitals Leuven, Leuven, Belgium; 5 Head, Exercise and Physical Activity Domain, Baker Heart and Diabetes Institute, 75 Commercial Rd, Melbourne, VIC 3004 Australia

**Keywords:** Diabetes, Exercise capacity, $\dot{V}O_{2}$peak, Exercise echocardiography, Diabetic cardiomyopathy, Diastolic dysfunction

## Abstract

**Background:**

The reasons for reduced exercise capacity in diabetes mellitus (DM) remains incompletely understood, although diastolic dysfunction and diabetic cardiomyopathy are often favored explanations. However, there is a paucity of literature detailing cardiac function and reserve during incremental exercise to evaluate its significance and contribution. We sought to determine associations between comprehensive measures of cardiac function during exercise and maximal oxygen consumption ($\dot{V}O_{2}$peak), with the hypothesis that the reduction in exercise capacity and cardiac function would be associated with co-morbidities and sedentary behavior rather than diabetes itself.

**Methods:**

This case–control study involved 60 subjects [20 with type 1 DM (T1DM), 20 T2DM, and 10 healthy controls age/sex-matched to each diabetes subtype] performing cardiopulmonary exercise testing and bicycle ergometer echocardiography studies. Measures of biventricular function were assessed during incremental exercise to maximal intensity.

**Results:**

T2DM subjects were middle-aged (52 ± 11 years) with a mean T2DM diagnosis of 12 ± 7 years and modest glycemic control (HbA_1c_ 57 ± 12 mmol/mol). T1DM participants were younger (35 ± 8 years), with a 19 ± 10 year history of T1DM and suboptimal glycemic control (HbA_1c_ 65 ± 16 mmol/mol). Participants with T2DM were heavier than their controls (body mass index 29.3 ± 3.4 kg/m^2^ vs. 24.7 ± 2.9, P = 0.001), performed less exercise (10 ± 12 vs. 28 ± 30 MET hours/week, P = 0.031) and had lower exercise capacity ($\dot{V}O_{2}$peak = 26 ± 6 vs. 38 ± 8 ml/min/kg, P < 0.0001). These differences were not associated with biventricular systolic or left ventricular (LV) diastolic dysfunction at rest or during exercise. There was no difference in weight, exercise participation or $\dot{V}O_{2}$peak in T1DM subjects as compared to their controls. After accounting for age, sex and body surface area in a multivariate analysis, significant positive predictors of $\dot{V}O_{2}$peak were cardiac size (LV end-diastolic volume, LVEDV) and estimated MET-hours, while T2DM was a negative predictor. These combined factors accounted for 80% of the variance in $\dot{V}O_{2}$peak (P < 0.0001).

**Conclusions:**

Exercise capacity is reduced in T2DM subjects relative to matched controls, whereas exercise capacity is preserved in T1DM. There was no evidence of sub-clinical cardiac dysfunction but, rather, there was an association between impaired exercise capacity, small LV volumes and sedentary behavior.

## Introduction

Exercise capacity is frequently reduced in people with diabetes mellitus (DM)—universally in those with type 2 diabetes mellitus (T2DM) but less consistently in people with type 1 DM (T1DM). Diabetic cardiomyopathy, described as cardiac dysfunction in the absence of coronary artery disease and cardiovascular risk factors such as hypertension [1, 2], has gained mainstream traction over the years as a unique pathophysiological entity affecting people with DM that may contribute to impaired exercise performance. A direct association between hyperglycemia, myocardial dysfunction and resulting congestive heart failure (CHF) has been reported [3] although this association seems to be much stronger for T2DM than for T1DM [4, 5]. These inconsistencies have led to some doubting the entity of diabetic cardiomyopathy altogether [6]. Indeed, there is overlap between risk factors for exercise intolerance and T2DM including sedentary behavior, obesity and resulting metabolic derangements. Rather than having a causal relationship, collinearity in risk factors may explain the association between diabetes and exercise intolerance, particularly in people with T2DM.

We sought to quantify exercise capacity in people with DM compared to healthy controls and establish the relative contribution of cardiac dysfunction using comprehensive measures of left ventricular (LV) and right ventricular (RV) function at rest and during exercise. Relative to healthy controls, we hypothesized that exercise capacity would be normal in T1DM and reduced in the T2DM sub-group, implying that metabolic comorbidities may provide an alternative explanation for reductions in exercise impairment and cardiac function.

## Research design and methods

### Subject recruitment

Subjects with T1DM and T2DM attending specialist hospital diabetes outpatient clinics were recruited via promotional leaflets and approval of their treating doctor. An equal number of those with- and without microvascular complications formed each group, defined by diabetic retinopathy, nephropathy and/or neuropathy according to current guidelines [7]. Inclusion criteria consisted of confirmed diabetes diagnosis, age 18–70 years, and sufficient physical capacity to perform low-intensity exercise.

A group of healthy controls were age- and sex- matched to each diabetes subgroup in a 2:1 ratio to balance demographic differences between the DM subgroups and allow inferences to be made when considering results for T1DM and T2DM groups. For both diabetes and control subjects, exclusion criteria included known coronary artery disease, resting LV systolic dysfunction (defined as LV ejection fraction < 40%), significant nephropathy (eGFR < 30 ml/min/1.73 m^2^), and chronic obstructive airways disease. Subjects were also excluded if echocardiographic images were non-diagnostic.

A detailed baseline assessment of microvascular and cardiovascular disease was performed in all subjects, including serum creatinine, urinalysis, retinal imaging for diabetic retinopathy grading, 24-h blood pressure monitoring, and echocardiography. History of diabetic neuropathy was assessed by review of each participant’s hospital medical record and diabetes outpatient clinic charts. A detailed diary of exercise habits over the 4 weeks prior to the study were recorded and the product of weekly exercise (hours) and intensity were used to quantify the metabolic equivalent (MET) hours.

### Exercise studies

Cardiopulmonary exercise testing was performed on an upright bicycle (Excaliber Sport, Lode, The Netherlands) using an individualized continuous incremental ramp protocol until exhaustion. Peak exercise capacity ($\dot{V}O_{2}$peak) was aimed to be reached within 10 min of exercise. Continuous 12-lead electrocardiography (ECG) monitored heart rate (HR), ST-segment changes and arrhythmia throughout exercise (Norav Medical, Israel) whilst blood pressure was recorded using an automated ECG-gated auscultatory device (Tango M2, SunTech Medical, USA). Breath-by-breath analysis of oxygen consumption and carbon dioxide production (JLab, CareFusion, Germany) was averaged over five breaths. Respiratory exchange ratio (RER), ventilatory threshold (VT) and ventilatory efficiency ($\dot{V}{\text{E}}/\dot{V}{\text{CO}}_{2}$) were calculated by standard measures [8].

Exercise echocardiography was completed on a semi-supine bicycle ergometer with lateral tilt (Lode, The Netherlands) over four exercise stages at increasing power calculated according to an individual’s $\dot{V}O_{2}$peak. We have demonstrated previously that 66% of maximal power obtained on an upright ergometer is equivalent to near-maximal intensity on a semi-supine ergometer [9]. Thus low, medium, high and maximal intensity exercise was prescribed as 15, 25, 50 and 66% of $\dot{V}O_{2}$peak respectively. After 1 min of commencing each exercise stage, image acquisition began with the aim to collect all images within 3 min. Breath holding was required in only a minority of studies predominantly in the latter stages of exercise to improve image quality, although a preference to extend the number of cardiac cycles recorded was favored. At rest and during each stage of exercise, apical four- and two-chamber, and parasternal long- and short-axis transthoracic images were collected to calculate comprehensive measures of biventricular systolic function according to guideline recommendations for performing exercise echocardiography [10]. Non-invasively derived central hemodynamic parameters including stroke volume (SV), cardiac output (CO), and pulmonary artery systolic pressure (PASP) were also calculated by standard measures [11]. The Doppler envelope of tricuspid regurgitation (TR) peak velocity was optimized by injection of 1–2 ml of an agitated colloid contrast to calculate PASP. Measurements of chamber volume, SV and CO were indexed to body surface area (BSA). Three to five beat loops were recorded for each 2D window while continuous wave (CW) and pulse wave (PW) Doppler recordings were extended to allow measurement and averaging of three beats. The highest recorded TR peak velocity was used for PASP at each level of exercise. Heart rate reserve was the difference of peak exercise and resting heart rate. All other measures of cardiac reserve were defined as the difference between peak exercise and resting measures as a percentage. Global longitudinal strain (GLS) was measured at rest using 2-dimensional speckle-tracking methods as described in detail previously [12]. Diastolic function was assessed at rest, and in early recovery after sufficient separation of the early (E) and late (A) diastolic mitral inflow waves. A single experienced cardiac sonographer conducted all studies using a Vivid E9 cardiac ultrasound machine (GE Healthcare), while one cardiologist with expertise in echocardiography analyzed all images offline using EchoPAC software (Version 113, GE Healthcare).

### Statistics

Data was analyzed using IBM SPSS Version 22 (SPSS Inc., Chicago, USA). Analysis of data normality was determined by the Shapiro–Wilk test. Continuous data was expressed as the mean value ± standard deviation (SD). A P < 0.05 was considered statistically significant. Differences between groups were assessed using unpaired t tests and Chi square Fisher’s exact test. Pearson’s coefficient evaluated univariate correlations. Two-way repeated measure ANOVA was used to assess the effect of diabetes and exercise stage on echocardiographic measures with changes during exercise analyzed as a within subjects factor and comparisons between groups as between subjects factors. A forward stepwise multiple linear regression was used to assess predictors of exercise capacity ($\dot{V}O_{2}$peak). Age, gender and BSA were included in the multiple regression as they are well described determinants of $\dot{V}O_{2}$peak. In addition, the strongest univariate predictors of $\dot{V}O_{2}$peak were included in the regression after excluding those variables demonstrating significant collinearity. Collinearity was considered significant when two factors were closely associated (R > 0.7). The number of variables entered into the model was restricted to 7, selected as a balance between including variables relevant to the study hypothesis whilst maintaining a reasonable balance for the cohort size.

## Results

Sixty-four subjects were recruited; three were excluded due to suboptimal echocardiography image quality (two with T1DM; one control) and one subject withdrew during the study. In total, 20 people with T1DM, 20 with T2DM, and 20 healthy age/sex-matched controls (10 matched to T1DM, 10 to T2DM) were included in the final analysis (Table 1).

**Table 1:** Patient demographics

	T1DM			T2DM		
	T1DM (n = 20)	Control to T1DM (n = 10)	P	T2DM (n = 20)	Control to T2DM (n = 10)	P
Age	35 ± 8	35 ± 9	0.99	52 ± 11	51 ± 13	0.8
Male (%)	13 (65)	6 (60)	0.74	16 (80)	7 (70)	0.66
Waist circumference (cm)	89 ± 13	84 ± 7	0.25	*103 *±* 10*	*91 *±* 9*	*0.005*
BMI (kg/m^2^)	25.7 ± 3.2	24.7 ± 3.4	0.44	*29.3 *±* 3.4*	*24.7 *±* 2.9*	*0.001*
BSA (m^2^)	1.9 ± 0.2	1.9 ± 0.2	0.8	2.1 ± 0.2	2.0 ± 0.2	0.25
HbA1c mmol/mol (%)	*65 *±* 16 (8.1 *±* 3.9)*	*33 *±* 2 (5.1 *±* 0.2)*	*< 0.0001*	*57 *±* 12 (7.4 *±* 1.1)*	*36 *±* 3 (5.4 *±* 0.2)*	<* 0.0001*
eGFR (ml/min/1.73 m^2^)	89 ± 4	88 ± 5	0.54	78 ± 17	87 ± 6	0.15
Urine ACR (mg/mmol)	1.4 ± 2.4	2.7 ± 4	0.30	9.7 ± 18.1	0.8 ± 0.6	0.13
Complications (%)	10 (50)			10 (50)		
Microalbuminuria (%)	3 (15)	0 (0)		8 (40)	0 (0)	
Retinopathy (%)	10 (50)	0 (0)		5 (25)	0 (0)	
Neuropathy (%)	2 (10)			1 (5)		
Diabetes duration (years)	19 ± 10			12 ± 7		
Diabetes therapy						
Insulin (%)	20 (100)			11 (55)		
Metformin (%)	0 (0)			19 (95)		
Sulfonylurea (%)	0 (0)			5 (25)		
GLP1/DPP4-I	0 (0)			7 (35)		
SGLT2-I	0 (0)			2 (10)		
ACE-I/ARB	4 (20)			11 (55)		
Statin	3 (15)	1 (10)	1.0	*14 (70)*	*2 (20)*	*0.019*
24 h average BP						
Systolic (mmHg)	121 ± 10	116 ± 11	0.21	130 ± 10	127 ± 12	0.53
Diastolic (mmHg)	74 ± 4	72 ± 6	0.33	79 ± 6	82 ± 9	0.27
Hypertension (%)	3 (15)	0 (0)	0.23	*10 (50)*	*1 (10)*	*0.049*
Smoker	0 (0)	0 (0)		3 (15)	0 (0)	0.06
MET hours	34.1 ± 30.5	40.2 ± 51.8	0.69	*10.5* ± *12.1*	*27.8 ± 30.0*	*0.031*
Cardiopulmonary test						
Maximum power (W)	236 ± 100	258 ± 89	0.57	*158 ± 46*	*230 ± 49*	*< 0.0001*
Power (% predicted)	120 ± 33	122 ± 26	0.87	*85 ± 18*	*115 ± 19*	*< 0.0001*
Maximum HR (bpm)	170 ± 15	179 ± 13	0.13	154 ± 21	165 ± 15	0.17
HR (% predicted)	92 ± 7	97 ± 5	0.07	92 ± 11	98 ± 8	0.15
$\dot{V}O_{2}$ (ml/min)	2886 ± 877	3238 ± 867	0.31	*2341 ± 601*	*2959 ± 521*	*0.01*
$\dot{V}O_{2}$peak (ml/min/kg)	38 ± 9	43 ± 13	0.20	*26 ± 6*	*38 ± 8*	*< 0.0001*
$\dot{V}O_{2}$peak (% predicted)	99 ± 20	112 ± 31	0.16	*88 ± 18*	*126 ± 33*	*< 0.0001*
$\dot{V}{\text{E}}/\dot{V}{\text{CO}}_{2}$	23 ± 3	22 ± 4	0.43	26 ± 3	23 ± 4	0.06
RER	1.23 ± 0.10	1.29 ± 0.11	0.17	1.20 ± 0.09	1.23 ± 0.08	0.44

Statistically significant differences are highlighted in italic

*BMI* body mass index, *BSA* body surface area, *HbA1c* glycated hemoglobin, *eGFR* estimated glomerular filtration rate, *ACR* albumin-creatinine ratio, *GLP1* glucagon-like peptide-1 receptor agonist, *DPP4-I* dipeptidyl peptidase-4 inhibitor, *SGLT2-I* sodium-glucose co-transporter-2 inhibitor, *ACE-I* angiotensin converting enzyme inhibitor, *ARB* angiotensin II receptor blocker, *BP* blood pressure, *MET* metabolic equivalent, *HR* heart rate, $\dot{V}O_{2}$ volume of oxygen, $\dot{V}O_{2}$*peak* peak oxygen consumption, $\dot{V}{\text{E}}/\dot{V}{\text{CO}}_{2}$ minute ventilation/volume of carbon dioxide, *RER* respiratory exchange ratio

The mean age of T1DM participants was 35 ± 8 years, predominantly male (n = 13, [65%]) and slightly overweight (BMI 25.7 ± 3.2 kg/m^2^), with a 19 ± 10-year history of T1DM and suboptimal glycemic control (HbA_1c_ 65 ± 16 mmol/mol). All T1DM subjects with microvascular complications had retinopathy, of which one had additional microalbuminuria, and two had microalbuminuria and neuropathy.

T2DM subjects were middle-aged (52 ± 11 years), male (n = 16, [80%]) and overweight (body mass index [BMI] 29.3 ± 3.4 kg/m^2^). The diagnosis of T2DM was made 12 ± 7 years previously, and current glycemic control was modest (HbA_1c_ 57 ± 12 mmol/mol). Microvascular complications comprised of isolated retinopathy in two (20%), isolated microalbuminuria in five (50%), retinopathy and microalbuminuria in two (20%) and combined retinopathy, microalbuminuria and neuropathy in one (10%).

While control groups were matched for age and sex, T2DM subjects were heavier than their controls (BMI 29.3 ± 3.4 vs. 24.7 ± 2.9 kg/m^2^, P = 0.001) with greater waist circumference (13 vs. 2% above normal upper limit reference for sex, P = 0.016), and performed less exercise (10 ± 12 vs. 28 ± 30 MET hours/week, P = 0.031). Similar differences were not present amongst T1DM and their controls.

### Cardiopulmonary exercise testing

**Fig. 1:**
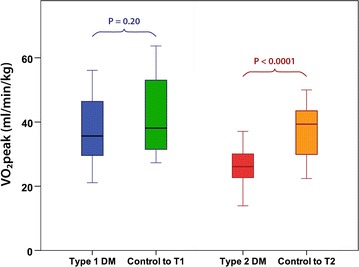
Lower $\dot{V}O_{2}$peak in subjects with T2DM relative to controls. Box plot graphs signifying the median, interquartile range (box) and minimum/maximum values (whiskers) for the comparison between T1DM and T2DM subjects relative to age and sex matched controls

$\dot{V}O_{2}$peak was significantly lower in T2DM subjects compared to their controls (Table 1 and Fig. 1). In contrast, those with T1DM had above-average fitness (power: 120 ± 33% predicted) and no significant difference in exercise capacity compared to their controls.

### Baseline echocardiography

Both DM groups had normal resting measures of biventricular systolic function and pulmonary pressures (Table 2). There was greater concentric remodeling amongst the T2DM cohort relative to their controls (55% vs. 10%; χ^2^ P = 0.024), because of lower LV end-diastolic volume (LVEDV) and a tendency to greater LV mass. There were no differences in cardiac structure (LV relative wall thickness, LV mass, LVEDV and left atrial volume) in T1DM subjects and their controls.

**Table 2:** Resting echocardiography measurements

	T1DM			T2DM		
	T1DM (n = 20)	Control to T1DM (n = 10)	P	T2DM (n = 20)	Control to T2DM (n = 10)	P
LVMI (g/m^2^)	78 ± 8	83 ± 13	0.19	78 ± 9	68 ± 18	0.07
RWT	0.38 ± 0.07	0.35 ± 0.06	0.35	*0.44 ± 0.07*	*0.36 ± 0.05*	*0.002*
LVEDVI (ml/m^2^)	50 ± 13	56 ± 19	0.28	*42* ± *10*	*54* ± *13*	*0.008*
LVEF (%)	60 ± 4	60 ± 5	0.93	59 ± 6	59 ± 5	0.91
LVs’ (cm/s)	6.5 ± 0.9	7.1 ± 1.1	0.11	5.8 ± 1.2	5.7 ± 1.0	0.76
LV GLS (%)	− 18.9 ± 2.4	− 19.9 ± 2.0	0.29	− 18.1 ± 2.1	− 19.8 ± 2.4	0.055
LV strain rate	− 1.1 ± 0.2	− 1.1 ± 0.1	0.45	− 1.1 ± 0.2	− 1.1 ± 0.2	0.93
LAVI (ml/m^2^)	34 ± 11	41 ± 10	0.13	*33* ± *6*	*39* ± *7*	*0.03*
RVs’ (cm/s)	10.5 ± 1.7	11.1 ± 1.5	0.33	10.0 ± 1.7	10.3 ± 1.2	0.56
RVFAC (%)	49 ± 5	46 ± 7	0.32	42 ± 4	45 ± 6	0.18
RV GLS (%)	− 29.2 ± 5.8	− 27.0 ± 4.3	0.31	− 24.9 ± 4.6	− 20.4 ± 13.4	0.19
PASP (mmHg)	24 ± 5	26 ± 4	0.45	29 ± 6	27 ± 4	0.33
E (m/s)	0.81 ± 0.16	0.77 ± 0.19	0.50	0.72 ± 0.13	0.66 ± 0.24	0.38
A (m/s)	0.49 ± 0.18	0.46 ± 0.12	0.63	*0.66* ± *0.15*	*0.45* ± *0.11*	*0.001*
E/A	1.8 ± 0.6	1.7 ± 0.6	0.77	*1.1* ± *0.3*	*1.6* ± *0.9*	*0.046*
DT (ms)	189 ± 22	192 ± 25	0.70	199 ± 28	200 ± 21	0.95
e’_sep_ (cm/s)	9.2 ± 1.8	9.3 ± 1.8	0.85	5.8 ± 1.6	6.9 ± 2.0	0.13
E/e’_sep_ (rest)	9 ± 3	9 ± 3	0.60	*13* ± *4*	*10* ± *2*	*0.02*
E/e’_sep_ (peak)	9 ± 2	8 ± 4	0.37	11 ± 2	10 ± 2	0.16

Statistically significant differences are highlighted in italic

*LVMI* left ventricular mass index, *RWT* relative wall thickness, *LVEDVI* left ventricular end diastolic volume index, *LVEF* left ventricular ejection fraction, *LVs’* LV tissue Doppler septal peak systolic velocity, *LV GLS* left ventricular global longitudinal strain, *LAVI* left atrial volume index, *RVs’* RV tissue Doppler free wall peak systolic velocity, *RVFAC* right ventricular fractional area change, *RV GLS* RV global longitudinal strain, *PASP* pulmonary artery systolic pressure, *E* early mitral inflow velocity, *A* late mitral inflow velocity, *DT* deceleration time, *e’*_*sep*_ tissue Doppler septal mitral annular early diastolic velocity, *E/e’*_*sep*_ ratio of early mitral inflow velocity to tissue Doppler septal mitral annular early diastolic velocity

The diastolic E/A ratio was lower and E/e’ ratio higher in T2DM subjects compared to their controls, while counter-intuitively left atrial volume index (LAVI) was lower. The prevalence of LV diastolic dysfunction according to current diagnostic guidelines was not different (20% of T2DM and control subjects alike; χ^2^ P = 1.0). No subject with T1DM had evidence of diastolic dysfunction.

### Exercise echocardiography

**Fig. 2:**
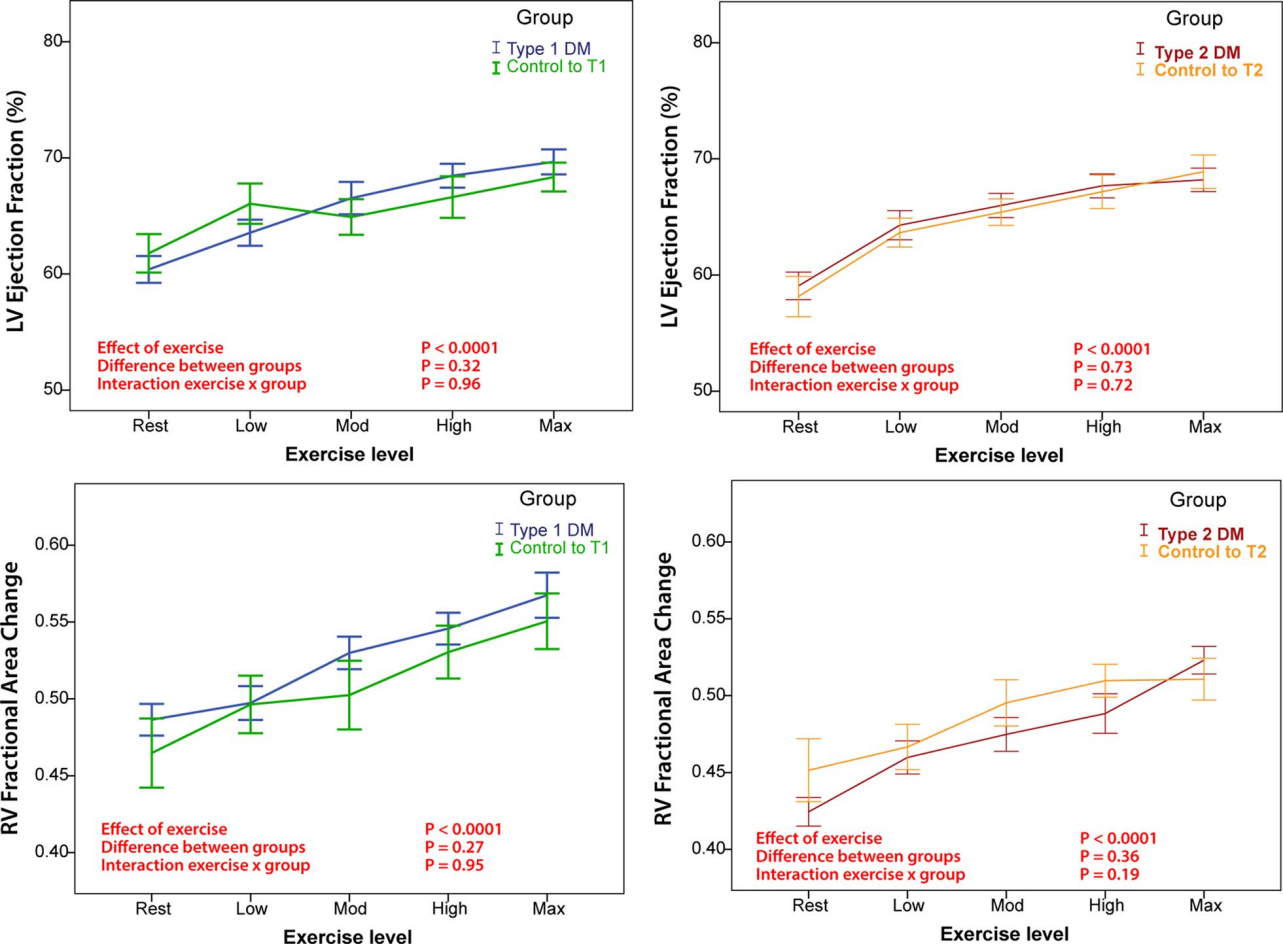
No difference in biventricular function in T1DM and T2DM relative to their matched controls. Comparisons between subjects with diabetes and control subjects demonstrate that there is significant augmentation in all measures during exercise (effect of exercise), but no difference in mean values during exercise (difference between groups) and no difference in the change in function during exercise (interaction exercise × group)

All measures of biventricular systolic function and hemodynamic parameters augmented significantly from resting to maximal exercise intensity in both DM groups (Table 3a, b and Fig. 2). No subject had evidence of inducible regional wall motion abnormalities on echocardiography or significant ischemic ECG changes.

**Table 3:** Repeated measures factorial ANOVA assessing cardiac reserve in type 1 (a) and type 2 (b) DM subjects

	Rest			Peak		
	T1DM	Control to T1DM	P value, baseline	Type 1 diabetes	Control to T1DM	P value*, interaction with exercise
(a) Type 1 DM subjects						
LVEF (%)	60 ± 5	62 ± 5	0.50	70 ± 5^†^	68 ± 4^†^	0.32
LVs’ (cm/s)	6.5 ± 0.9	7.1 ± 1.1	0.11	10.6 ± 1.8^†^	11.5 ± 1.9^†^	0.12
LVEDVI (ml/m^2^)	52 ± 12	61 ± 14	0.11	46 ± 13^†^	56 ± 15	0.10
RVFAC (%)	49 ± 5	46 ± 7	0.32	57 ± 7^†^	55 ± 6^†^	0.86
RVs’ (cm/s)	10.5 ± 1.8	11.1 ± 1.5	0.33	17.1 ± 2.3^†^	18.3 ± 1.8^†^	0.55
PASP (mmHg)	24 ± 5	26 ± 4	0.45	53 ± 11^†^	53 ± 5^†^	0.27
CI (l/min/m^2^)	2.1 ± 0.3	2.5 ± 0.6	*0.024*	5.2 ± 1.0^†^	6.2 ± 1.3^†^	0.43
HR (bpm)	67 ± 11	66 ± 12	0.93	140 ± 17^†^	145 ± 9^†^	0.57
SVI (ml/m^2^)	32 ± 6	38 ± 7	*0.021*	38 ± 8^†^	43 ± 9^†^	0.43

	Rest			Peak		
	T2DM	Control to T2DM	P value, baseline	T2DM	Control to T2DM	P value*, interaction with exercise
(b) Type 1 DM subjects						
LVEF (%)	59 ± 5	58 ± 6	0.66	68 ± 5^†^	69 ± 4^†^	0.73
LVs’ (cm/s)	5.8 ± 1.2	5.7 ± 1.0	0.76	8.6 ± 1.5^†^	9.6 ± 2.1^†^	0.08
LVEDVI (ml/m^2^)	49 ± 10	58 ± 16	0.07	46 ± 8^†^	55 ± 14	*0.96*
RVFAC (%)	42 ± 4	45 ± 6	0.18	52 ± 4^†^	51 ± 4^†^	0.28
RVs’ (cm/s)	10.0 ± 1.7	10.3 ± 1.2	0.56	16.1 ± 2.8^†^	16.7 ± 1.8^†^	0.66
PASP (mmHg)	29 ± 6	27 ± 4	0.33	57 ± 10^†^	54 ± 7^†^	0.23
CI (l/min/m^2^)	2.3 ± 0.5	2.3 ± 0.5	0.94	4.9 ± 1.0^†^	5.9 ± 1.4^†^	*0.001*
HR (bpm)	73 ± 13	65 ± 9	0.11	125 ± 18^†^	130 ± 14^†^	*0.001*
SVI (ml/m^2^)	32 ± 6	35 ± 4	0.19	40 ± 7^†^	45 ± 8^†^	0.60

Values are mean ± SD

Statistically significant differences between DM and control groups are highlighted in italic

*LVEF* left ventricular ejection fraction, *LVs’* LV tissue Doppler septal peak systolic velocity, *LVEDVI* LV end diastolic volume index, *RVFAC* right ventricular fractional area change, *RVs’* RV tissue Doppler free wall peak systolic velocity, *PASP* pulmonary artery systolic pressure, *CI* cardiac index, *HR* heart rate, *SVI* stroke volume index

* P value represents the comparison between regressions of multiple measures during exercise with slope coefficients compared between diabetes and control subjects

^†^P < 0.0001 for peak exercise vs. baseline for individual groups

### Cardiac function during incremental exercise stages

#### T1DM

**Fig. 3:**
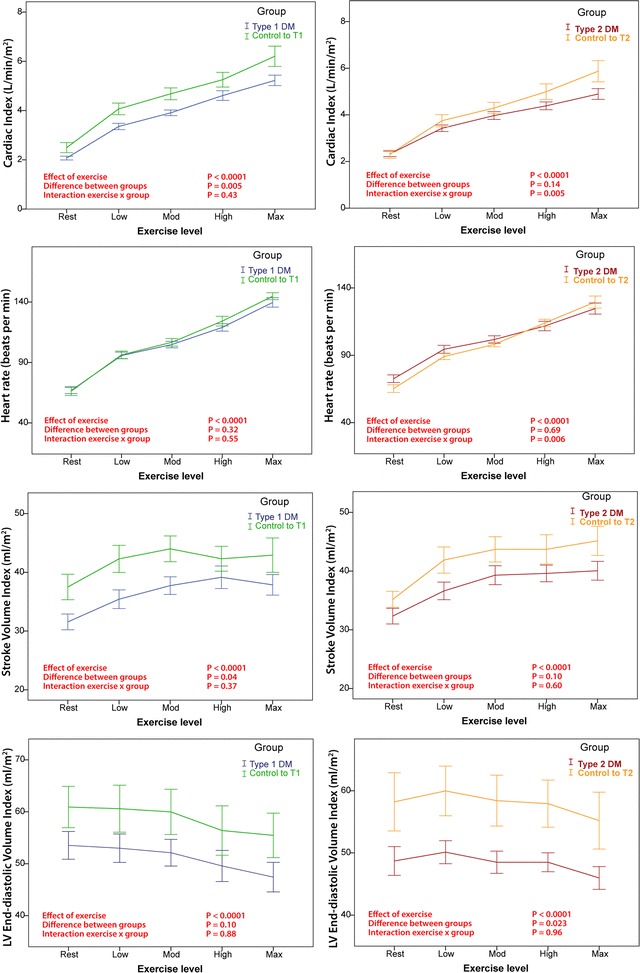
Differences in cardiac index, heart rate, stroke volume and end-diastolic volume in diabetic and control groups. In T1DM subjects there was a slightly lower stroke volume index and cardiac index as compared with matched controls, but the augmentation of these measures was similar between groups (interaction exercise × group, P > 0.05). As compared with matched controls, the increase in cardiac index was less in T2DM (interaction exercise × group, P = 0.005) due to a lesser increase in heart rate (interaction exercise × group, P = 0.006)

LVEF augmentation with exercise was similar in T1DM and control subjects (Table 3a, Fig. 2). LV septal myocardial systolic velocity (LVs’) was lower in T1DM throughout exercise (mean LVs’: 8.5 ± 0.2 vs. 9.4 ± 0.3 cm/s; P = 0.029), but the rate of increase during exercise stages was similar. Throughout exercise, T1DM subjects had 13% lower stroke volume index (SVI) and 16% cardiac index throughout exercise (P = 0.04 and P = 0.005 respectively) but the degree of augmentation with exercise was similar (P = 0.37 and P = 0.44 for the interaction respectively), see Fig. 3. There was no significant difference in the pattern of LVEDV-index (LVEDVI) reduction during exercise compared to their controls.

#### T2DM

Augmentation of cardiac index was greater in matched controls than T2DM subjects (P = 0.005 for interaction), due to a significantly greater heart rate reserve in controls (P = 0.006), Fig. 3. There were no differences in measures of LV or RV systolic function to contribute to this observation (see Fig. 2). Resting LVEDVI tended to be lower in the T2DM group (49 ± 10 vs. 58 ± 16, P = 0.07) and was overall 16% lower throughout exercise in T2DM as compared with controls (P = 0.023). However, the change in LVEDVI was similar in both groups during exercise (interaction between LVEDVI and exercise, P = 0.96). There were no differences in systemic blood pressure and pulmonary artery pressure augmentation between groups.

### Diastolic stress test

E/e′ post exercise was surprisingly normal in all four subjects identified to have LV diastolic dysfunction at rest. Only one subject in the study cohort—with T2DM—met criteria (E/e′ > 15) for LV diastolic dysfunction at peak exercise.

### Predictors of exercise performance

**Fig. 4:**
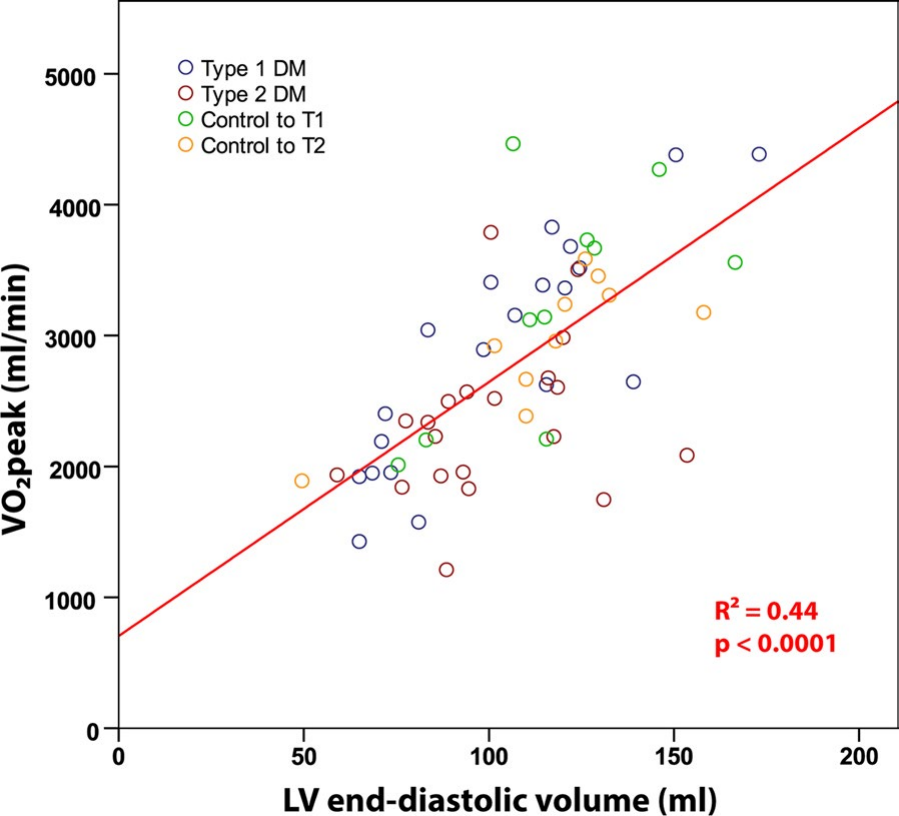
Correlation between cardiac volumes and exercise capacity. The scatter graph and Pearson’s correlation demonstrate a moderately strong correlation in which LV end-diastolic volume accounts for 44% of the variance in $\dot{V}O_{2}$ peak

Significant univariate correlates of $\dot{V}O_{2}$peak are presented in Table 4. The strongest univariate associations with $\dot{V}O_{2}$peak were LVEDV (accounting for 44% of variance, see Fig. 4), RV end-diastolic area (RVEDA), MET-hours of physical activity and cardiac index reserve.

**Table 4:** Univariate correlates of $\dot{V}O_{2}$peak (ml/min)

Variable	r	P
Female sex	− 0.45	< 0.0001
T2DM	− 0.39	0.002
HbA1c (mmol/mol)	− 0.37	0.004
hsCRP (mg/l)	− 0.32	0.013
Age	− 0.30	0.021
LVEDV (ml)	0.67	< 0.0001
RVEDA (cm^2^)	0.58	< 0.0001
MET-hour equivalents	0.57	< 0.0001
CI-reserve (%)	0.55	< 0.0001
LV mass (g)	0.43	0.001
HR-reserve (bpm)	0.40	0.002
LVs’-reserve (%)	0.38	0.003
Hemoglobin (g/l)	0.35	0.006
BSA (m^2^)	0.32	0.013
RVFAC reserve (%)	0.30	0.022

*HbA1c* glycated hemoglobin, *hsCRP* high sensitivity C-reactive protein, *LVEDV* left ventricular end diastolic volume, *RVEDA* right ventricular end diastolic area, *CI-reserve* cardiac index reserve, *HR-reserve* heart rate reserve, *LVs’-reserve* LV tissue Doppler septal peak systolic velocity reserve, *BSA* body surface area, *RVFAC* right ventricular fractional area change

A multivariate analysis was performed to predict $\dot{V}O_{2}$peak from a selection of the significant univariate correlates. Age, sex and BSA accounted for 49% of the variance in $\dot{V}O_{2}$peak (Table 5a). After adjustment for the above three factors, significant positive predictors of $\dot{V}O_{2}$peak were LVEDV and estimated MET-hours of physical activity, whilst T2DM was a negative predictor (Table 5b). The combined model accounted for 80% of the variance in $\dot{V}O_{2}$peak (R^2^ = 0.80; P < 0.0001).

**Table 5:** Multivariate predictors of VO_2_ (ml/min)—model 1 and 2

Variable	Coefficient (B)	SE	95% CI	P
(a) Model 1				
Intercept	1623.1	931.6		
Age	− 34.6	7.0	− 48.8 to − 20.5	< 0.0001
Sex (female)	− 846.4	189.3	− 1226.3 to − 466.5	< 0.0001
BSA	1494.1	486.1	518.6 to 2469.6	0.003
(b) Model 2				
Intercept	− 258.6	647.6		
Age	− 18.7	5.5	− 29.8 to − 7.7	0.001
Sex (female)	− 532.3	130.3	− 794.1 to − 270.5	< 0.0001
BSA	1719.6	354.2	1007.9 to 2431.3	< 0.0001
MET hours	8.0	2.0	4.1 to 12.0	< 0.0001
T2DM	− 471.2	133.4	− 739.4 to − 203.0	0.001
LVEDV	6.2	2.3	1.7 to 10.8	0.008

*B* unstandardized regression coefficient, *SE* standard error of the coefficient, *Standardized B* standardized coefficient, *95% CI* 95% confidence interval, *BSA* body surface area, *LVEDV* LV end diastolic volume

## Discussion

To our knowledge this is the first and largest study assessing comprehensive measures of cardiac and hemodynamic function at rest and during incremental exercise to maximal exertion as determinants of exercise capacity in people with T1DM and T2DM, relative to matched controls. We observed lower $\dot{V}O_{2}$peak in T2DM subjects and determined that 80% of the variance in $\dot{V}O_{2}$peak in the overall cohort was explained by cardiac size (lower resting LVEDV), the amount of habitual exercise and T2DM, in addition to demographic factors age, sex and BSA. Conversely, there was no evidence of lower exercise performance in the T1DM cohort despite a lengthy duration of diabetes and suboptimal glycemic control. In both diabetes subgroups, there was no evidence of biventricular systolic dysfunction or impaired LV diastolic function at rest or during exercise relative to their matched controls.

### Hyperglycemia does not explain reductions in $\dot{V}O_{2}$peak

Chronic hyperglycemia is associated with endothelial dysfunction and microvascular disease, and a causal link with congestive heart failure (CHF) and reduced exercise capacity [2] has been proposed. In contrast to previous investigations [13, 14], we observed a significant inverse correlation between HbA1c and $\dot{V}O_{2}$peak in our cohort. However, this association was largely abolished when other confounding factors such as diabetic status, age and sex were included in the multivariate model.

It may be argued that the associations between hyperglycemia and exercise capacity are best explored in T1DM rather than T2DM subjects given the lower prevalence of other confounding factors such as obesity, additional cardiovascular risk factors and lower exercise participation. Most studies have failed to identify a clear link between glycemic control and exercise capacity [15, 16], with the exception of Baldi et al. [17]. Baldi compared a group of 12 T1DM endurance triathletes with matched controls and whilst there was no difference in exercise capacity, the 6 T1DM athletes with worse glycemic control (mean HbA1c = 62 mmol/mol) had 15% lower $\dot{V}O_{2}$peak than the six athletes with better control (mean HbA1c = 48 mmol/mol). The degree to which these differences can be accounted for by hyperglycemia as opposed to confounding behavioral and health factors is difficult to quantify. The data presented here suggests that once multiple factors are considered, the influence of glycemic control on exercise capacity is, at most, modest.

### An absence of subclinical cardiac dysfunction

A higher incidence of congestive heart failure (CHF) has been observed in diabetes subjects [18, 19], leading to the concept of a diabetes-specific cardiomyopathy [2]. Diabetic cardiomyopathy has evolved into a specific clinical entity with two distinct phenotypes proposed: heart failure with preserved ejection fraction (HFpEF) and heart failure with reduced ejection fraction (HFrEF) [2]. Furthermore, Widya et al. [20] have reported changes in the right ventricle that parallel those seen in the left ventricle in T2DM subjects. On the other hand, the entity of diabetic cardiomyopathy remains contentious amongst some given the dependence of small animal and molecular models rather than prospective human data [6, 21].

A high prevalence of LV diastolic dysfunction has been observed in T2DM [22, 23] and its association with impaired exercise capacity has been suggested to represent a subclinical phase of diabetic cardiomyopathy [24, 25]. In the present study, we found no difference in the prevalence of diastolic dysfunction between the T2DM group and their controls. Interestingly, the four T2DM subjects identified to have diastolic dysfunction at rest had normal diastolic stress test results. Similarly, there were no differences in the change of LVEDV during exercise when DM subjects were compared with matched controls (Fig. 3). These findings strengthen the argument that diastolic dysfunction was not the cause for the reduced $\dot{V}O_{2}$peak.

There were also no significant differences in LV or RV systolic function to explain the lower $\dot{V}O_{2}$peak. Subjects with reduced LVEF were excluded from participating in our study, and the mean LVEF in the total cohort was 60 ± 5%. Reductions in newer echocardiographic measures of LV systolic function such as global longitudinal strain (GLS) [26–28] and tissue Doppler myocardial velocity [29], however, can identify subclinical LV systolic dysfunction in the presence of a normal ejection fraction. In our cohort of T2DM subjects there was a non-significant trend to reduced resting LV GLS. However, LV GLS did not correlate with $\dot{V}O_{2}$peak amongst T2DM subjects (R = − 0.08, P = 0.78), and other echocardiography measures of LV function had similarly poor associations with exercise capacity. During exercise, contractile reserve, as quantified by LVEF and LVs’ augmentation, was similar and normal in both the T2DM group and controls.

### Reduced augmentation of cardiac index in T2DM

It has been demonstrated previously that resting cardiac index is similar in healthy individuals regardless of fitness level [30], and thus the similar resting cardiac index across the groups in our cohort was expected. However, in T2DM subjects the augmentation of cardiac index during exercise was reduced, explained by significant differences in heart rate but not stroke volume.

### Lower heart rate reserve contributes to reduced cardiac index in T2DM

Heart rate reserve was significantly reduced in those with T2DM compared to controls (mean 52 ± 13 bpm vs. 64 ± 11 bpm; P = 0.018) due to both a higher resting heart rate and a lower peak exercise heart rate.

Possible explanations for the difference in heart rate reserve include diabetic cardiovascular autonomic neuropathy (CAN), reduced β-adrenoreceptor sensitivity or remodeling of the sinoatrial node. It is possible that the reduced HR reserve could also reflect a relative lack of physical exercise conditioning. Only one of our T2DM participants had a diagnosis of diabetic neuropathy, which is perhaps less than may be expected [31, 32]. There is a large variability in the reported prevalence of diabetic CAN [33], although it appears to affect a similar proportion of people with type 1 and T2DM [34]. We note that our T1DM cohort displayed several risk factors for CAN including lengthy duration of DM, suboptimal glycemic control and additional microvascular disease. We therefore contend that should we have underestimated the number of subjects with CAN, both T1DM and T2DM groups should have been affected equally. This is not supported by our findings of reduced exercise capacity only in the T2DM group and not in those with T1DM.

Wilson et al. [35, 36] recently reported an almost identical reduction in HR reserve in T2DM subjects as compared with control subjects. They investigated whether this may be attributable to reduced β-adrenoreceptor sensitivity. However, the β-adrenoreceptor agonist dobutamine was associated with a greater relative increase in HR in T2DM subjects as compared with controls suggesting that factors other than β-adrenoreceptor dysfunction were responsible. It is possible that diabetes is associated with remodeling of ion-channels within the sino-atrial node that affect rate control, but this has not previously been investigated.

On the other hand, exercise conditioning may explain the differences in heart rate reserve. Habitual exercise is associated with greater heart rate reserve, mainly because of lower resting heart rate [30] consistent with the observations in this study. Regular exercise programs, including high intensity interval training (HIIT), have been repeatedly shown to improve a range of cardiovascular measures and outcomes in people with DM, including lower resting heart rates and improved heart rate reserve [37–44]. Thus, it is reasonable to argue that the observation in our study that heart rate reserve was reduced in T2DM, but not in T1DM, may be explained by prior exercise conditioning rather than by diabetes itself.

### No differences in SVI, LVEDVI and LVESVI in DM subjects vs. controls

The most definitive demonstration that cardiac function did not differ between DM subjects and matched controls was the demonstration that cardiac volumes changed similarly during exercise. There were no differences in SVI, LVEDVI or LVESVI during incremental exercise between T2DM or T1DM and their respective control groups. Resting LVEDVI was lower in T2DM subjects and remained so throughout incremental exercise (16% overall, P = 0.023). As illustrated in Fig. 3, the LVEDVI change mirrored that of controls (interaction of exercise and group, P = 0.96) suggesting that there was no impairment in diastolic filling. This is consistent with a recent study by Wilson et al. [35] who also found no difference between changes in LVEDV during exercise in T2DM as compared with controls. Furthermore, the pattern of change in cardiac volumes during exercise in our study are the same as those described for normal physiology using exercise echocardiography [45] and exercise cardiac magnetic resonance imaging [9, 46]. We contend that the anatomy of the LV itself (smaller LVEDV) may be the major factor contributing to attenuated cardiac reserve during exercise, rather than impaired diastolic filling of a small LV cavity.

### Increased LV volume predicts greater exercise capacity

It has been well demonstrated that exercise capacity correlates strongly with cardiac size (LV volumes, LV mass or whole heart volumes), both in athletic and non-athletic populations [30, 47, 48]. We previously demonstrated that in the absence of overt contractile dysfunction, differences in cardiac function during exercise were modest and thus the major determinant of stroke volume during intense exercise was stroke volume at rest [30]. In the current study, we found no evidence of LV systolic or diastolic dysfunction and, like the experience in athletes, found strong correlations between resting cardiac geometry measures (LV mass, LVEDV and RVEDA) and $\dot{V}O_{2}$peak. LVEDV was most strongly associated with $\dot{V}O_{2}$peak in the entire cohort and remained a significant independent predictor on multivariate analysis after adjusting for age, sex and BSA. Most literature to date has focused on measures of cardiac function to explain exercise capacity in diabetes. We could not identify differences in function despite comprehensive resting and exercise measures. On the other hand, we identified differences in cardiac size (LVEDV) and we found a robust correlation between LVEDV and $\dot{V}O_{2}$peak. This is perhaps not surprising given that cardiac output is a major determinant of exercise capacity and, in the absence of differences in cardiac function, the major determinant of stroke volume is cardiac size.

It is notable, however, that we did not find a strong relationship between LVEDV and $\dot{V}O_{2}$peak in all sub-groups. Whilst the association in the combined group of T1DM, T2DM and controls was strong, the correlation was diminished when comparing T2DM and controls or T1DM and controls (R = 0.51, P = 0.004 and R = 0.79, P < 0.0001, respectively). These comparisons are challenged by the limitations of sample size and also by the influence of covariates. Simple correlations do not consider the important confounders of age, gender and BSA, all of which influence $\dot{V}O_{2}$peak. Thus, the more robust analysis is the multivariate analysis in which LVEDV remained a significant independent predictor of $\dot{V}O_{2}$peak.

### Limited activity may explain exercise performance

It is generally reported that levels of physical activity in people with T2DM are less than that of the general non-diabetic population [49, 50] and this was also true in our comparison between T2DM subjects and controls according to estimated MET-hours. More importantly, weekly exercise participation was a significant independent predictor of $\dot{V}O_{2}$peak on multivariate analysis.

The question arises as to how $\dot{V}O_{2}$peak could diminish in the setting of preserved cardiac function and how this could relate to reduced physical activity? Haykowsky et al. elegantly demonstrated that sedentary behavior was associated with reductions in $\dot{V}O_{2}$peak that was related to fatty infiltration and atrophy of peripheral muscle, thereby compromising peripheral O_2_ diffusive metabolism [51]. Similarly, Russell et al. [52] attributed differences in exercise capacity between T2DM and controls to changes in the peripheral vasculature and muscle interface. We can only hypothesize by inference from our data as we did not assess the quality of peripheral muscle gas exchange.

**Fig. 5:**
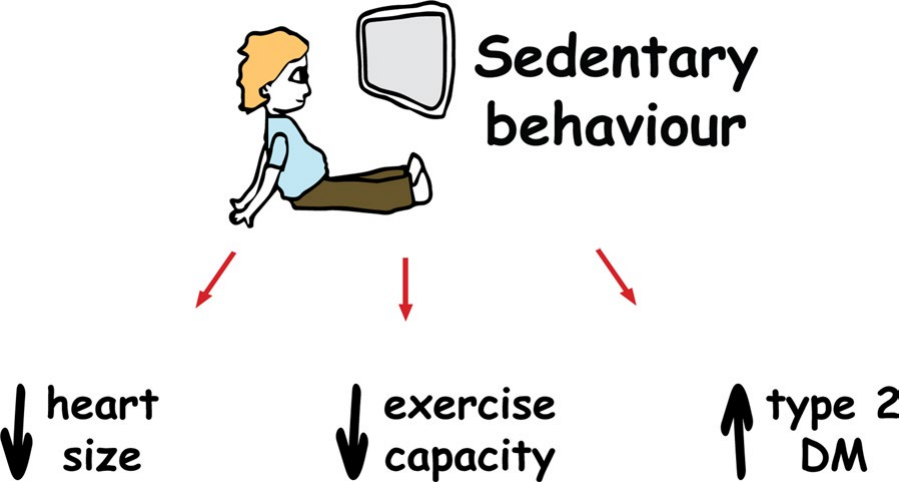
Collinearity between exercise capacity, heart size and T2DM. The lack of relationship between reduced exercise capacity and T1DM as well as the strong association with sedentary behavior and T2DM suggests that sedentary behavior may be the common link between T2DM and reduced exercise capacity

T2DM is most prevalent in older adults, and aging is associated with a linear decline in exercise capacity, accelerating after age 50 [53]. Physical inactivity compounds this age-related decline in $\dot{V}O_{2}$peak and increases the risk of cardiovascular mortality in people with T2DM [54]. The Dallas Bed Rest and Exercise Study [55] eloquently described a loss of cardiorespiratory fitness, cardiac mass and ventricular volumes that occurred following 3 weeks of bed rest. This extreme intervention resulted in cardiovascular changes equivalent to 30 years of aging. It has also been reported that one of the strongest predictors of heart failure in older adults is their level of fitness two decades earlier [56], and thus chronic inactivity could result in cardiac atrophy and reductions in functional capacity whilst also increasing insulin resistance and diabetes risk. As summarized in Fig. 5, our observed association between less physical activity, smaller cardiac volumes and T2DM, lends weight to the premise that inactivity could be the common underlying factor causing both diabetes and exercise intolerance.

## Clinical implications

It has been suggested that cardiac limitation in diabetes relates to increased fibrosis and stiffness of the myocardium [2]. Typical heart failure treatments have not proved efficacious and novel therapies have primarily targeted anti-fibrotic pathways. Such treatments may continue to prove fruitless if the cause of exercise limitation in diabetes is predominantly due to relative cardiac atrophy (or lack of physiological hypertrophy) associated with a sedentary lifestyle. Exercise is one of the few efficacious treatments in heart failure with preserved ejection fraction [57] and this may be because it directly addresses the causative mechanisms. Our data identifies a strong association between sedentary behavior and reduced functional capacity implying that exercise may prove the best therapy for the prevention and treatment of exercise intolerance in patients with diabetes.

## Limitations

The risk of recruitment bias is challenging to avoid in an exercise study given the tendency for healthier subjects to volunteer for a study that includes exercise assessment. However, DM subjects were recruited from specialist outpatient clinics where patients tend to have higher rates of co-morbidities than direct community recruitment. Patient demographics, glycemic control and medication use were similar to those described in previous DM cohorts suggesting that a healthy cohort bias does not explain the lack of cardiac dysfunction identified in the study. DM subjects were not excluded if known to have hypertension, which is a confounder to the diagnosis of diabetic cardiomyopathy. Given that half of the T2DM group had a pre-existing diagnosis of hypertension, we contend that the normal measures of cardiac reserve strengthen our finding that myocardial dysfunction and therefore diabetic cardiomyopathy was not the cause of reduced exercise capacity.

Although our DM group was equally split into T1DM and T2DM, we elected not to perform direct comparisons between these DM subtypes other than to perform univariate and multivariable analyses of the entire cohort. In our institution, it would not have been feasible to match T1DM and T2DM participants for age without significantly altering disease duration and the presence and severity of diabetic complications between the two groups, based on the demographics of our institution’s DM clinic patients. However, we believe the use of smaller healthy control groups to each DM subtype allows indirect comparisons to be drawn, in addition to collating all subject data for multivariable analyses.

We relied on hospital records to determine the presence of diabetic neuropathy and did not perform cardiovascular autonomic reflex tests. This may have led to an underestimation of the number of DM subjects affected by mild diabetic neuropathy and CAN. However, our DM subjects had relatively mild severity of microvascular complications despite longstanding diabetes, and had no difference in resting heart rate or sinus tachycardia to suggest significant CAN.

Invasive measurements of central hemodynamics were not performed and beyond the scope of the current study design. Nonetheless it has previously been demonstrated that Doppler echocardiography-derived estimates of CO and PASP allow for accurate measurements with moderate precision [58].

Finally, we did not quantify the degree of hyper-insulinemia and its effect on cardiovascular or peripheral vascular function. Insulin resistance is associated with reduced exercise capacity in heart failure populations with [59, 60] and without [61] diabetes. However, as hyper-insulinemia is also present in obesity and metabolic syndrome, the ability to discern the relative contribution of obesity and diabetes is difficult and we do not think would alter the interpretation of our findings.

## Conclusions

In our cohort of DM subjects, T2DM was associated with reduced exercise capacity whereas active subjects with T1DM had preserved exercise capacity relative to healthy controls. Reduced physical activity and smaller LV volumes, rather than subclinical cardiac dysfunction, were associated with impaired exercise capacity. These findings suggest that physical inactivity may be a stronger predictor of exercise intolerance than hyperglycemia or myocardial dysfunction, and a powerful reminder to encourage exercise prescription to people with T2DM.

## Abbreviations

BSA: body surface area
CAN: cardiovascular autonomic neuropathy
CHF: congestive heart failure
CO: cardiac output
CW: continuous wave
DM: diabetes mellitus
ECG: electrocardiography
HFpEF: heart failure with preserved ejection fraction
HFrEF: heart failure with reduced ejection fraction
HIIT: high intensity interval training
HR: heart rate
LAVI: left atrial volume index
LV: left ventricle
LVEDV: left ventricular end-diastolic volume
LVEF: left ventricular ejection fraction
LVESV: left ventricular end-systolic volume
LV GLS: left ventricular global longitudinal strain
PASP: pulmonary artery systolic pressure
PW: pulse wave
RVEDA: right ventricular end-diastolic area
SV: stroke volume
T1DM: type 1 diabetes mellitus
T2DM: type 2 diabetes mellitus
$\dot{V}O_{2}$peak: oxygen consumption at peak exercise
